# Epitope-tagged and phosphomimetic mouse models for investigating natriuretic peptide-stimulated receptor guanylyl cyclases

**DOI:** 10.3389/fnmol.2022.1007026

**Published:** 2022-10-19

**Authors:** Jeremy R. Egbert, Tracy F. Uliasz, Katie M. Lowther, Deborah Kaback, Brandon M. Wagner, Chastity L. Healy, Timothy D. O’Connell, Lincoln R. Potter, Laurinda A. Jaffe, Siu-Pok Yee

**Affiliations:** ^1^Department of Cell Biology, University of Connecticut Health Center, Farmington, CT, United States; ^2^Center for Mouse Genome Modification, University of Connecticut Health Center, Farmington, CT, United States; ^3^Department of Integrative Biology and Physiology, University of Minnesota, Minneapolis, MN, United States; ^4^Department of Biochemistry, Molecular Biology, and Biophysics, University of Minnesota, Minneapolis, MN, United States

**Keywords:** natriuretic peptide receptor, guanylyl cyclase, phosphorylation, genetically modified mice, cyclic GMP

## Abstract

The natriuretic peptide receptors NPR1 and NPR2, also known as guanylyl cyclase A and guanylyl cyclase B, have critical functions in many signaling pathways, but much remains unknown about their localization and function *in vivo*. To facilitate studies of these proteins, we developed genetically modified mouse lines in which endogenous NPR1 and NPR2 were tagged with the HA epitope. To investigate the role of phosphorylation in regulating NPR1 and NPR2 guanylyl cyclase activity, we developed mouse lines in which regulatory serines and threonines were substituted with glutamates, to mimic the negative charge of the phosphorylated forms (NPR1-8E and NPR2-7E). Here we describe the generation and applications of these mice. We show that the HA-NPR1 and HA-NPR2 mice can be used to characterize the relative expression levels of these proteins in different tissues. We describe studies using the NPR2-7E mice that indicate that dephosphorylation of NPR2 transduces signaling pathways in ovary and bone, and studies using the NPR1-8E mice that indicate that the phosphorylation state of NPR1 is a regulator of heart, testis, and adrenal function.

## Introduction

Natriuretic peptide receptors 1 and 2 (NPR1 and NPR2) are membrane guanylyl cyclases, also known as guanylyl cyclase A and guanylyl cyclase B (Potter, 2011; Kuhn, 2016). Atrial natriuretic peptide (ANP) and brain natriuretic peptide (BNP) activate NPR1 to reduce blood pressure and cardiac hypertrophy and to regulate metabolism (Kuhn, 2016; Wagner et al., 2022a). C-type natriuretic peptide (CNP) activates NPR2 to regulate bone growth and oocyte meiosis (Kuhn, 2016; Jaffe and Egbert, 2017; see sections “Results and discussion”), as well as axon bifurcation (Schmidt et al., 2018, 2022) and cardiovascular remodeling and contractility (Kuhn, 2016; Subramanian et al., 2018).

**FIGURE 1:**
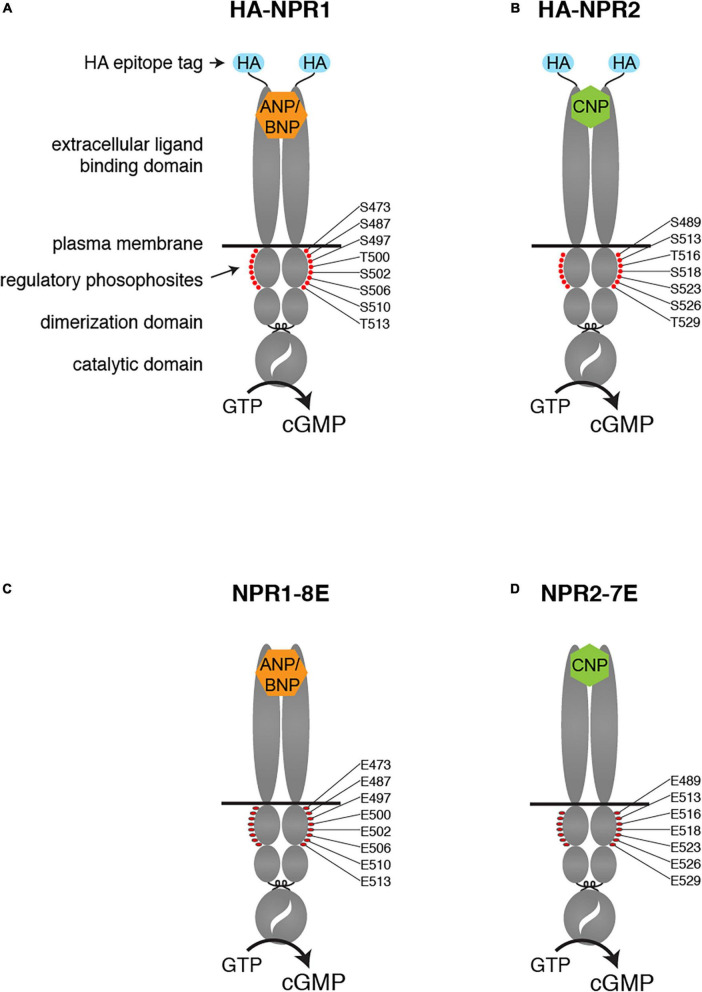
Modifications of the NPR1 and NPR2 proteins in HA-NPR1, HA-NPR2, NPR1-8E, and NPR2-7E mice. Diagrams show the domains of each protein, including the HA epitope tag at the N-terminus **(A,B)** and the glutamate substitutions for regulatory phosphosites within the kinase homology domain **(C,D)**.

NPR1 and NPR2 are structurally similar proteins, comprised of an extracellular ligand binding domain, a single plasma membrane-spanning region, and an intracellular domain composed of a juxtamembrane kinase homology domain, followed by a dimerization domain and a carboxy-terminal catalytic domain (Figures 1A,B). The kinase homology domain of these receptors does not appear to possess intrinsic kinase activity (Edmund et al., 2019) but is highly phosphorylated on multiple serines and threonines by unidentified kinases. Importantly, phosphorylation of the kinase homology domain is required to transduce the natriuretic peptide binding signal from the extracellular domain to the catalytic domain (Potter and Hunter, 1998a,b). Hence, dephosphorylation of these receptors provides a direct mechanism for inhibiting natriuretic peptide-dependent cellular cGMP generation, and thus opposing cGMP-dependent signaling pathways.

Tryptic phosphopeptide mapping and mass spectrometry studies identified the phosphorylation sites in NPR1 and NPR2 (Potter and Hunter, 1998a,b, 1999; Schröter et al., 2010; Yoder et al., 2010). Substitution of alanines to mimic dephosphorylated residues for known phosphorylated serine and threonine residues resulted in reduced natriuretic peptide-dependent guanylyl cyclase activity in transfected cells and genetically modified mice (Potter and Hunter, 1998a,b; Schmidt et al., 2018). Conversely, substitution of glutamates to constitutively mimic the negative charge of the phosphates resulted in the NPR1-8E and the NPR2-7E enzymes that are activated like the phosphorylated wild-type enzymes but cannot be inactivated by dephosphorylation (Yoder et al., 2012; Otto et al., 2017).

Much has been learned about the physiological functions of NPR1 and NPR2 from studies of natural mutations in humans and genetically modified mice in which these proteins are inactive or overactive (Oliver et al., 1997, 1998; Bartels et al., 2004; Tamura et al., 2004; Zhang et al., 2010; Miura et al., 2012; Pandey, 2019), and in mice expressing cyclic GMP biosensors (Götz et al., 2014; Subramanian et al., 2018; Feil et al., 2022). In this report, we describe several recently developed mouse models in which NPR1 and NPR2 are epitope-tagged or have phosphomimetic mutations. Because endogenous expression levels of natriuretic peptide-stimulated receptor guanylyl cyclases are relatively low, antibodies that work well for studies of NPR1 and NPR2 in overexpressing cells do not always have sufficient sensitivity and specificity to be useful for native tissues. To address this issue, we generated mice with influenza hemagluttinin (HA) epitope tags on NPR1 and NPR2 (Baena et al., 2020, and results to be described; Figures 1A,B).

To extend knowledge of how phosphorylation of these proteins regulates their activity in transfected cells to understanding of physiological functions *in vivo*, we generated mice in which the 7 serines and threonines of NPR1 and the 6 serines and threonines in NPR2 that are known to be phosphorylated were changed to glutamates (Figures 1C,D). We also converted to glutamate one additional serine that is highly conserved between guanylyl cyclases that are regulated by phosphorylation (Ser-473 in NPR1 and Ser-489 in NPR2). These modifications produced phosphomimetic forms of each receptor called NPR1-8E and NPR2-7E. These glutamate-substituted forms are activated by natriuretic peptides like the phosphorylated wild-type receptors but cannot be inactivated by dephosphorylation, resulting in consequences for multiple physiological systems (Shuhaibar et al., 2016, 2017; Egbert et al., 2018; Robinson et al., 2020; Wagner et al., 2021, 2022a,2022b).

This report describes how mice expressing HA-tagged and glutamate-substituted NPR1 and NPR2 were produced, and summarizes published studies using them. We also present new findings that have been obtained with these mice, which we hope will facilitate future studies.

## Methods

### Animal studies

Protocols covering the generation of mice, maintenance of mouse lines and subsequent use of the animals were approved by the Institutional Animal Care Committees at the University of Connecticut Health Center and the University of Minnesota. Mice were housed in ventilated cages and racks and kept under a 12/12 h or 14/10 h light/dark cycle. They were provided with standard mouse chow and water *ad libitum*.

### Generation of mice with hemagluttinin tags on NPR1 and NPR2

To generate the HA-NPR1 and HA-NPR2 mouse lines, a 9-amino acid HA epitope tag (YPYDVPDYA) was added to the extracellular N-termini of the endogenous NPR1 and NPR2 proteins (Figures 1A,B), by CRISPR/Cas9 mediated genome editing of C57BL/6J embryos. Generation and validation of the HA-NPR2 mice have been previously described (Baena et al., 2020). We also made mice in which FLAG or PA epitope tags were used in place of the HA tag on NPR2, but these mice did not show specific labeling on western blots or immunofluorescence using available antibodies (Baena et al., 2020).

**FIGURE 2:**
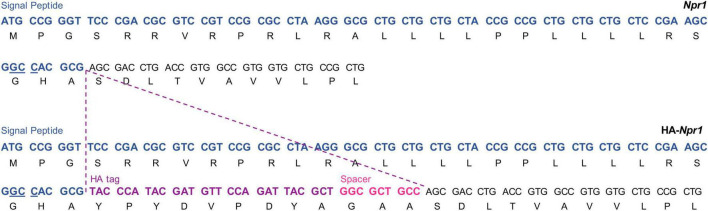
Generation of the HA-NPR1 mouse strain using CRISPR. The Npr1 signal peptide sequence is shown in blue with the PAM site for CRISPR underlined. The HA tag sequence is shown in purple, and the sequence of the 3 amino acid spacer is shown in pink. The Npr1 exon 1 coding sequence is shown in black.

The HA-NPR1 mice were made similarly to the HA-NPR2 mice, except that the Cas9/sgRNA ribonucleoprotein (RNP) complex and ssDNA donor mixture were introduced directly into one-cell C57BL/6J embryos by electroporation rather than microinjection. The positions where the HA sequence and a 3 amino acid spacer were inserted are shown in Figure 2. The spacer is aimed to introduce structural flexibility to improve antibody binding efficiency as well as to minimize any interference of ligand binding. DNA sequences for generation and genotyping of these mice are listed in Supplementary Table 1.

The mice used for the studies to be described were homozygous for the indicated mutations, and the terms HA-NPR1 and HA-NPR2 refer to homozygotes. The mice used to generate them were obtained originally from The Jackson Laboratory (#000664).

### Generation of mice in which the regulatory serines and threonines of NPR1 and NPR2 are substituted by glutamates

Generation and validation of the NPR1-8E and NPR2-7E mice have been previously described (Shuhaibar et al., 2016; Wagner et al., 2022a). In these lines, 8 serines and threonines of NPR1, and 7 serines and threonines in NPR2, were changed to glutamates (Figures 1C,D). The terms NPR1-8E and NPR2-7E refer to homozygotes that express the glutamate mutations globally.

In brief, the NPR1-8E mice were produced by CRISPR/Cas9 mediated genome editing of C57BL/6J one-cell embryos, to obtain a global knock-in line; they were initially named GC-A-8E (Wagner et al., 2022a). The mice were maintained on the C57BL/6J background.

The NPR2-7E mice were generated by homologous recombination in mouse ES cells derived from a F1 (C57BL/6Jx129sv) embryo, to obtain a conditional knock-in line (Shuhaibar et al., 2016). To remove the Neo cassette and obtain a globally expressing line, the conditional knock-in line was then bred with C57BL/6J *Hprt-Cre* mice (Tang et al., 2002). The *Hprt-Cre* mice had been previously backcrossed for >20 generations with C57BL/6J mice. The background of the resulting NPR2-7E global knock-in line was C57BL/6J (75%)/129sv (25%). The mice were maintained on this mixed background. Use of heterozygous breeding pairs to obtain homozygous mutants and wildtype controls ensured that background strain did not affect experimental results (Shuhaibar et al., 2016, 2017). In some studies, the NPR2-7E mice were further backcrossed with C57BL/6J mice (Egbert et al., 2018; Robinson et al., 2020; Wagner et al., 2021).

### Western blotting

Tissues for western blots were collected from HA-NPR1 or HA-NPR2 homozygous mice, or wild-type controls. Ovaries were obtained from 24-day old female mice that had been injected ∼44 h prior to collection with 5 IU equine chorionic gonadotropin (ProSpec #HOR-272) to stimulate follicle growth. All other tissues were collected from adult male mice (16–20 weeks old). After removal, organs were rinsed in PBS, blotted on a laboratory wipe, and snap frozen in liquid N_2_. Frozen organs were weighed and sonicated in an appropriate volume of 50 mM Tris HCl, 150 mM NaCl, 1% SDS; protein concentrations were determined by a BCA assay (Thermo Scientific #23227). Protein samples were loaded on a 12% gel, and separated by SDS-PAGE, allowing the 45 kDa marker to run to the bottom of the gel. Loading of equal amounts of protein per lane was assured by performing BCA assays on all tissues, and confirmed by staining of blots for total protein, using Ponceau S (Sander et al., 2019) (Fisher Scientific #BP103-10) (Supplementary Figure 1). Blots were probed with a rabbit monoclonal antibody against the HA epitope (Cell Signaling Technology #3724).

Several commercially available HA tag antibodies were tested, and those from Cell Signaling resulted in the most specific signal. Depending on the application, either the mouse monoclonal (#2367) or rabbit monoclonal (#3724) antibody from Cell Signaling was found to be optimal (Baena et al., 2020; Egbert et al., 2021; Shuhaibar et al., 2021).

### Measurement of ejection fraction and end systolic volume in hearts of aged NPR1-8E and wild-type mice

These parameters were measured as previously described (Wagner et al., 2022a). In brief, echocardiography was performed on isoflurane-anesthetized mice using the Vevo 2100 (FujiFilm VisualSonics Inc., Toronto, Canada) with a MS550 transducer. Echocardiographic images were captured as mice were recovering from anesthesia. At the time of imaging, heart rates for all groups were 400–500 bpm (Supplementary Table 2).

## Results and discussion

### Use of hemagluttinin-NPR1 and hemagluttinin-NPR2 mice to investigate tissue and cell expression patterns

**FIGURE 3:**
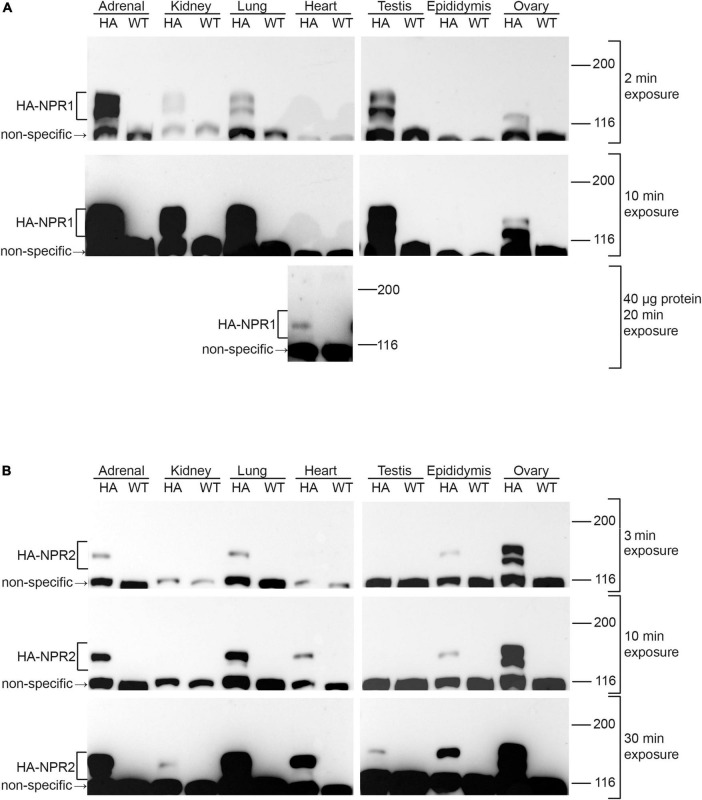
Western blots of NPR1 and NPR2 proteins in native tissues from mice expressing HA-NPR1 and HA-NPR2. Tissue samples from homozygous HA-NPR1 **(A)** and HA-NPR2 **(B)** mice were prepared similarly (see section “Methods”). Except as indicated, 20 μg of protein was loaded per lane (determined by a BCA assay). Three exposures of each blot are shown. Bands corresponding to NPR1 and NPR2 are seen only in the samples from the mice with the HA tags, not in those from wild-type mice. Doublet bands at ∼120 and 130 kDa represent differentially glycosylated and phosphorylated forms (Koller et al., 1993; Dickey et al., 2016; Shuhaibar et al., 2016). A non-specific band is present at ∼110 kDa. The figure shows only the top half of the blots, where NPR1 and NPR2 are located; the complete range of molecular weights for the blots in **(A)** is shown in Supplementary Figure 1A. Supplementary Figures 1B,C shows corresponding Ponceau stained blots and densitometry measurements, confirming that equal amounts of protein were loaded per lane.

To investigate the utility of the HA-tagged NPR1 and NPR2 mice for comparing expression levels of NPR1 and NPR2 proteins, lysates of several tissues were prepared from homozygous HA-tagged and wild-type mice, and an equal amount of protein from each tissue lysate was separated by SDS-PAGE. Western blots were probed with an HA antibody, and bands present at ∼120–130 kDa in HA-expressing tissues, but not in wild-type tissues, were identified as NPR1 or NPR2 (Figure 3). Doublet bands at ∼120 and 130 kDa represent differentially glycosylated and phosphorylated forms (Koller et al., 1993; Dickey et al., 2016; Shuhaibar et al., 2016).

Of the tissues tested, NPR1 was most highly expressed in the adrenal gland and testis (Figure 3A; 2 min exposure), whereas NPR2 was most highly expressed in ovary (Figure 3B; 3 min exposure). In addition to its presence in the adrenal gland and testis, NPR1 protein was detected at lower levels in kidney, lung, and ovary (Figure 3A; 2 and 10 min exposures). However, NPR1 was only detected in heart with more total protein loaded per lane and a longer exposure of the blot, indicating a relatively low expression level (Figure 3A; 40 μg protein, 20 min exposure). Consistently, although NPR1 activity is found in heart membranes, its activity in heart is only ∼10% of that in kidney (Wagner et al., 2022a). In addition to its presence in ovary, NPR2 was detected in adrenal gland, lung, heart, and epididymis (Figure 3B; 3 and 10 min exposures), and with a longer exposure of the blot, faint bands were seen in kidney and testis (Figure 3B; 30 min exposure). Previous western blot studies using HA-NPR2 mice have also demonstrated high NPR2 expression in primary chondrocytes (Shuhaibar et al., 2021).

Only a small subset of tissues was examined here, and previous studies indicate that many other tissues (including nerves, vasculature, and adipocytes) express these proteins as well (Kuhn, 2016; Schmidt et al., 2018, 2022). Because antibodies that are highly specific for NPR1 and NPR2 are not commercially available, mice expressing HA-tagged NPR1 and NPR2 will be useful for future studies evaluating protein expression levels in other tissues.

HA-NPR2 mice and HA antibodies have also been used to analyze the cellular localization of NPR2 within ovarian follicles, by immunofluorescence microscopy (Baena et al., 2020). These studies confirmed that NPR2 protein is present in granulosa cells, but not in the oocyte. Within the granulosa compartment, fluorescence measurements showed that the concentration of NPR2 is higher in the cumulus cells compared to the mural granulosa cells, and allowed a quantitative comparison of the amounts in each compartment (Baena et al., 2020). Immunofluorescence microscopy using HA-tagged NPR1 and NPR2 could also be useful for investigating the cellular distribution of these proteins in other complex tissues.

In addition to their use for examining protein expression levels in different tissues, HA-NPR2 mice and HA specific antibodies have been used together with Phostag gel western blots to show that fibroblast growth factor causes NPR2 dephosphorylation in chondrocytes (Shuhaibar et al., 2021). Similar methods using HA-NPR2 mice also showed that luteinizing hormone causes NPR2 dephosphorylation in ovarian follicles (Egbert et al., 2021), confirming previous studies using ovarian follicles from wild-type rats and an antibody made against an NPR2 peptide (Egbert et al., 2014).

Importantly, no obvious morphological or physiological defects have been noted with the HA-NPR1 and HA-NPR2 mice. A previous study demonstrated that addition of an epitope tag of similar size (FLAG, DYKDDDDK) to the N-terminus of NPR1 had no effect on the ANP concentration required to activate NPR1 guanylyl cyclase activity, or on the kinetics of the activation (Schröter et al., 2010). The N-terminal FLAG tag also had no effect on the expression level or subcellular localization of the NPR1 protein (Schröter et al., 2010). These findings for N-terminally FLAG-tagged NPR1, together with the evidence for the normal function of N-terminally HA-tagged NPR2 discussed below, support the conclusion that the HA-tag on NPR1 is unlikely to perturb its function.

A previous study demonstrated that addition of an HA tag to the N-terminus of NPR2 did not alter physiological processes that depend on NPR2 activity: regulation of meiotic arrest and resumption in ovarian follicles (Baena et al., 2020), and dephosphorylation of NPR2 in ovarian follicles and in chondrocytes in response to hormone or growth factor signaling (Egbert et al., 2021; Shuhaibar et al., 2021). In addition, the cumulative numbers of pups weaned from both HA-NPR2 and HA-NPR1 homozygous breeding pairs were similar to the numbers of pups weaned from wild-type pairs (Baena et al., 2020 for HA-NPR2, and similar results for HA-NPR1). Thus the presence of the HA tags on NPR1 and NPR2 N-termini did not appear to impair the function of these proteins.

### Use of NPR1-8E and NPR2-7E mice to investigate physiological functions of NPR1 and NPR2 phosphorylation

Analysis of the effects of substituting glutamates for the juxtamembrane phosphorylated serines and threonines of NPR1 and NPR2 in cells *in vitro* identified multiple phosphorylation sites in NPR1 and NPR2 that are important for regulation of enzyme activity (Potter and Hunter, 1998a,b, 1999; Schröter et al., 2010; Yoder et al., 2010, 2012; Otto et al., 2017). Subsequently, mice homozygous for these same mutations were generated, allowing determination of the functional consequences of the NPR2-7E mutations in ovarian follicles (Shuhaibar et al., 2016; Egbert et al., 2018), growing bones (Shuhaibar et al., 2017; Wagner et al., 2021), and adult bones (Robinson et al., 2020; Wagner et al., 2022b). Likewise, the NPR1-8E mutations were found to alter cardiac size and function, as well as plasma steroid and creatinine levels (Wagner et al., 2022a). Here we summarize what has been learned from these mice, and present new results about cardiac function in aging mice.

Importantly, previous studies have indicated that the NPR1-8E or NPR2-7E mutations have no effect on the ANP or CNP concentrations required to activate guanylyl cyclase activity (Shuhaibar et al., 2016; Otto et al., 2017) or on circulating plasma ANP, BNP, or CNP concentrations (Wagner et al., 2021, 2022b). The similar EC_50_ values for natriuretic peptide-dependent cyclic GMP generation indicate that the mutations do not affect ANP or CNP binding to their receptors. The 8E and 7E mutations also do not affect the amount of NPR1 and NPR2 protein in tissues from these mice (Shuhaibar et al., 2016, 2017; Wagner et al., 2022a).

### Function of NPR2 phosphorylation state in control of meiotic progression in oocytes within ovarian follicles

In mammalian preovulatory ovarian follicles, cGMP produced by NPR2 in the somatic (granulosa) cells diffuses through gap junctions to the oocyte, where it prevents meiotic resumption (Norris et al., 2009; Zhang et al., 2010; Robinson et al., 2012; Geister et al., 2013; Shuhaibar et al., 2015). The mid-cycle surge of luteinizing hormone (LH) restarts oocyte meiosis largely through the rapid dephosphorylation and inactivation of NPR2, which lowers cGMP levels in the granulosa cells, and by way of gap junctions, lowers cGMP in the oocyte as well (Egbert et al., 2014, 2021; Shuhaibar et al., 2015). This model was conclusively tested with follicles from NPR2-7E/7E mice, which maintain elevated cGMP levels in the presence of LH (Shuhaibar et al., 2016; Egbert et al., 2018). In NPR2-7E/7E follicles, LH-induced oocyte meiotic resumption is delayed by 5 h, although it eventually occurs due to other parallel, but slower, signaling mechanisms (Shuhaibar et al., 2016).

### Function of NPR2 phosphorylation state in control of bone length, mass, and strength

In chondrocytes of the growth plate in the bones of young mice, cGMP produced by CNP stimulation of NPR2 promotes bone elongation (Yasoda et al., 1998). Bones are shorter in mice and people with mutations that inactivate NPR2 (Bartels et al., 2004; Tamura et al., 2004; Tsuji and Kunieda, 2005; Khan et al., 2012; Geister et al., 2013; Nakao et al., 2015; Schmidt et al., 2018). Conversely bone length is increased by mutations that result in increased NPR2 activity in the absence of CNP (Miura et al., 2012, 2014; Hannema et al., 2013).

One pathway opposing bone elongation is fibroblast growth factor (FGF) signaling, which dephosphorylates and inactivates NPR2 in chondrocytes (Robinson et al., 2017; Shuhaibar et al., 2021), lowering cGMP levels in the growth plate (Shuhaibar et al., 2017, 2021). However, in growth plates from mice globally expressing NPR2-7E/7E, FGF has no effect on NPR2 activity, indicating that NPR2 dephosphorylation contributes to FGF signaling in chondrocytes (Shuhaibar et al., 2017). Correspondingly, NPR2-7E/7E mice have longer bones compared to wild-type mice (Shuhaibar et al., 2017; Wagner et al., 2021). Importantly, in mice with an activating mutation of the human FGF receptor 3 (FGFR3-G380R), which is a model of the congenital form of short stature known as achondroplasia (Lee et al., 2017), bone growth is rescued when the FGFR3-G380R mice are crossed with NPR2-7E/7E mice (Wagner et al., 2021). This suggests that a key part of the mechanisms that cause achondroplasia is the ability of FGFR3 activation to promote the dephosphorylation of NPR2. A phosphatase inhibitor that opposes this dephosphorylation increases bone elongation in a mouse model of achondroplasia (Shuhaibar et al., 2021).

In addition to these effects on bone elongation, the bones of NPR2-7E/7E mice have higher mineral density, a larger network of trabeculae, a thicker cortex, and larger diameter compared to wild type (Robinson et al., 2020). As a result of these features, NPR2-7E/7E tibias and femurs have elevated stiffness, toughness, and maximum load before fracture compared to wild type. Thus, NPR2-7E/7E mice not only have longer bones, but also increased bone mass and bone strength, as a result of increased osteoblasts as well as decreased osteoclasts (Robinson et al., 2020; Wagner et al., 2022b).

### Function of NPR1 phosphorylation state in control of heart size and contractility

**FIGURE 4:**
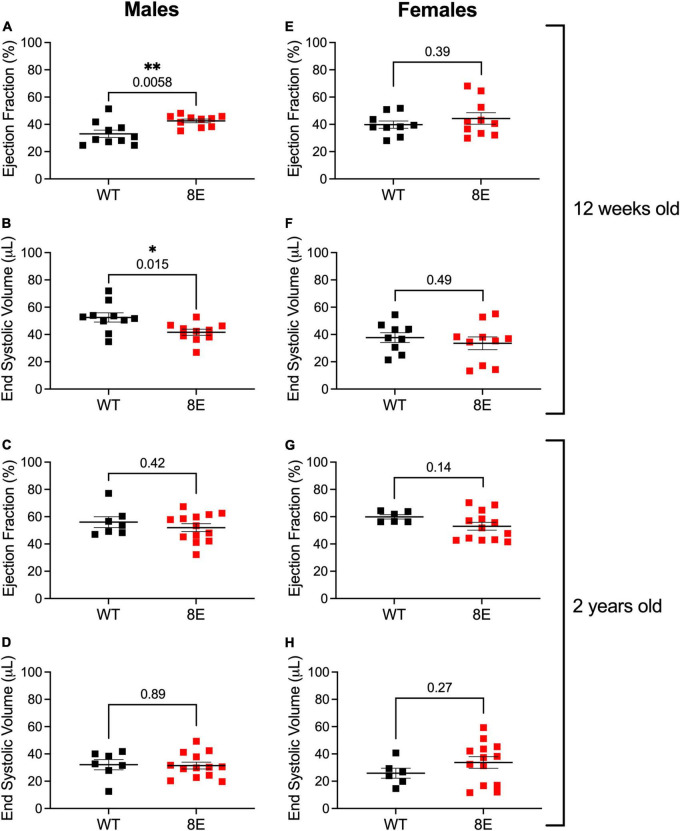
Unlike 12-week-old NPR1-8E male mice, 2-year-old NPR1-8E male mice do not have increased ejection fractions or decreased end systolic volumes. 12-week-old **(A,B,E,F)** and 2-year old **(C,D,G,H)** male and female mice were assessed by echocardiography for ejection fractions and end systolic volumes as described by Wagner et al. (2022a). Data for NPR1-WT and NPR1-8E mice are shown by black and red symbols, respectively. Long horizontal bars indicate the mean and vertical bars indicate SEM. Statistical differences were determined using a two-tailed student’s *t*-test with associated *p*-values shown in each panel; asterisks indicate significant differences *p < 0.05 and **p < 0.01. Heart rates at the time of imaging were the same for all groups (Supplementary Table 2). **(A,B,E,F)** Reproduced, as permitted by the publisher, from Wagner et al. (2022a).

Twelve-week-old male NPR1-8E/8E mice have smaller hearts. This is due to smaller cardiomyocytes, resulting from diminished cardiac extracellular regulated kinase (ERK) activity (Wagner et al., 2022a). Importantly, the smaller hearts have improved systolic function, as indicated by increased ejection fraction and decreased end systolic volume (Wagner et al., 2022a; Figures 4A,B). These findings indicate that NPR1 phosphorylation is a regulator of heart size and contractility. However, heart weight, ejection fraction, and end systolic volume in 12-week-old female mice were unaffected by the 8E mutations, despite similar levels of NPR1 protein in female and male mice (Wagner et al., 2022a; Figures 4E,F). Ejection fraction and end systolic volume were also unaffected by the 8E mutations in 2-year-old male or female mice (Figures 4C,D,G,H). Nevertheless, the hearts from the 2-year-old male NPR1-8E mice (*n* = 9) remained significantly smaller than hearts from age-matched wild-type male mice (*n* = 5) (*p* = 0.0002; two-tailed *t*-test).

Because NPR1 is expressed in multiple tissues, and at relatively low levels in the heart itself (Figure 3A and Wagner et al., 2022a), the effects of the 8E mutations on cardiac size and function, and their gender and age dependence, could have multiple causes. A direct effect of the phosphorylation state of NPR1 in the heart (Holtwick et al., 2003), which could be affected by adrenergic or other agonists, is one possible mechanism; systemic effects of elevated aldosterone and testosterone (Wagner et al., 2022a), could also contribute. Future studies of mice with tissue specific NPR1-8E mutations could help to identify the tissues in which NPR1 phosphorylation state acts to regulate cardiac function. Likewise, mice with HA-tagged NPR1 could be used to investigate the age dependence of NPR1 expression in the heart, which could contribute to understanding of why NPR1 phosphorylation state is a regulator of heart function only in young males. Similar studies of heart function in NPR2-7E mice would also be informative, and mice with HA-tagged NPR1 and NPR2 could be useful for analysis of the cellular and subcellular distribution of these proteins in the heart (Subramanian et al., 2018). Furthermore, these mice could be used to test whether inputs from the autonomic nervous system alter the phosphorylation state of NPR1 or NPR2 in the heart or other tissues (see below).

### Function of NPR1 phosphorylation state in control of steroid generation in testis and adrenal gland

Correlating with the high levels of expression of NPR1 in testis and adrenal gland (Figure 3), plasma levels of steroids produced by these tissues, testosterone and aldosterone, respectively, are elevated in young male NPR1-8E/8E mice (Wagner et al., 2022a). Correspondingly, luteinizing hormone (LH) receptor agonists, and the NPR1 agonists ANP and BNP, elevate cGMP and stimulate production of testosterone in Leydig cells from rat testis (Andric et al., 2007). Thus the elevation of testosterone in NPR1-8E/8E mice suggests that LH stimulation of testosterone production could result in part from phosphorylation of NPR1. In future studies, HA-NPR1 mice and Phostag gel western blot analysis could be used to investigate if NPR1 phosphorylation in testis is increased in response to LH. In the adrenal gland, the stimulation of aldosterone production in zona glomerulosa cells by adrenocorticotropic hormone and by angiotensin II, is cGMP-dependent (Gambaryan et al., 2003), suggesting that NPR1 phosphorylation might also contribute to the action of these agonists. If LH, ACTH, and/or AngII do increase NPR1 phosphorylation, the phosphorylation state of NPR1 could be investigated as a possible mediator of hormone-stimulated testosterone and aldosterone synthesis. Similarly, autonomic inputs to these tissues could lead to changes in NPR1 phosphorylation, possibly revealing another facet of steroid regulation.

### Summary and future directions

The discovery 25 years ago that the guanylyl cyclase activity of natriuretic peptide receptors requires receptor phosphorylation (Potter and Hunter, 1998a,b, 1999) raised the question of how this regulatory mechanism might function physiologically. Studies using the genetically modified mice described here have identified hormonal signaling pathways that act by decreasing phosphorylation of NPR2 in ovary and bone (Shuhaibar et al., 2016, 2017, 2021; Egbert et al., 2018, 2021; Robinson et al., 2020; Wagner et al., 2021, 2022b). Recent studies using these mice have suggested that related mechanisms may regulate physiological signaling in other organs such as the heart, testis and adrenal gland (Wagner et al., 2022a). Future studies using these and related mouse lines may help to elucidate these functions.

## Data availability statement

Cryopreserved embryos for the mouse lines described here are freely available by request: HA-NPR1 (global), HA-NPR2 (global), PA-NPR2 (global), NPR1-8E (global), NPR2-7E (global), and NPR2-7E (conditional). Requests should be addressed to S-PY (syee@uchc.edu).

## Ethics statement

This animal study was reviewed and approved by Institutional Animal Care and Use Committees at UCONN Health and University of Minnesota.

## Author contributions

S-PY, KL, and DK designed, generated, and validated the genetically modified mice. JE, TU, and LJ designed, performed, and analyzed the protein expression studies. BW, CH, TO’C, and LP designed, performed, and analyzed the heart measurements. JE, LP, S-PY, and LJ wrote the manuscript. All authors reviewed and approved it.
