# Supplementary material for: Epitope-tagged and phosphomimetic mouse models for investigating natriuretic peptide-stimulated receptor guanylyl cyclases
Source: Front Mol Neurosci. 2022 Oct 19;15:1007026. doi: 10.3389/fnmol.2022.1007026 (PMC9627482; doi:10.3389/fnmol.2022.1007026)
Supplement: Supplementary file 1 [file Data_Sheet_1.docx]

Supplementary Material

**Supplementary Table** **1.** DNA sequences for generation and genotyping of HA-NPR1 mice.

| **Type of sequence** | **Name** | **Sequence** |
| --- | --- | --- |
|  |  |  |
| sgRNA | *Npr1* | 5’- CCA CGG TCA GGT CGC TCG CG |
| ssDNA donor# | *Npr1* | 5’-A*T*G* CCG GGT TCC CGA CGC GTC CGT CCG CGC CTA AGG GCG CTG CTG CTG CTA CCG CCG CTG CTG CTG CTC CGA AGC GGC CAC GCG TAC CCA TAC GAT GTT CCA GAT TAC GCT GGC GCT GCC AGC GAC CTG ACC GTG GCC GTG GTG CTG CCG CTG ACC AAC ACC TCG TAC CCG* T*G |
| Primer§ | *Npr1* E1A | 5’-CCC GAG GAC GGC GAT CAG ACC ATG |
|  | *Npr1* E1R | 5’-GCC TTC ACC CTC CCG AGA GC |

# Asterisks denote phosphorothioate linkages, used to prevent exonuclease degradation.

# § The *Npr1* E1A and *Npr1* E1R primer pair was designed to amplify fragments of 252 bp and 288 bp, specific for NPR1 wildtype and HA-NPR1 alleles, respectively. This primer pair was also used for PCR followed by sequencing for genotype confirmation.

**Supplementary Table 2.** Heart rate of male and female wild type or *Npr1*-8E/8E mice at two different ages during collection of echocardiographic data shown in Fig. 4. Values are presented as mean heart rate (bpm) ± standard deviation for the number of mice shown in parentheses.

| **Mouse age** | **WT/WT Male** | **8E/8E Male** | **WT/WT Female** | **8E/8E Female** |
| --- | --- | --- | --- | --- |
| 12 weeks# | 438 ± 40  (10) | 434 ± 52  (10) | 435 ± 53  (9) | 436 ± 56  (10) |
| 2 years | 473 ± 27  (7) | 431 ± 33  (13) | 457 ± 40  (6) | 458 ± 34  (13) |

# 12-week data are reproduced, as permitted by the publisher, from Wagner et al. (2022a).

**Supplementary figure 1**. A) Full blot images for the top panel of figure 3A. B) Ponceau S-stained images for the blot shown in A. C) Densitometry of the Ponceau S-stained images shown in B. The total protein stain intensity for each lane was measured using ImageJ software (https://imagej.nih.gov/ij/download.html).
